# Healthy Community-Life Circle Planning Combining Objective Measurement and Subjective Evaluation: Theoretical and Empirical Research

**DOI:** 10.3390/ijerph19095028

**Published:** 2022-04-21

**Authors:** Jiangjun Wan, Yutong Zhao, Kaili Zhang, Chunchi Ma, Haiying Sun, Ziming Wang, Hongyu Wu, Mingjie Li, Lingqing Zhang, Xiaohong Tang, Ying Cao, Li Tang, Jinxiu Yang

**Affiliations:** 1 School of Architecture and Urban-Rural Planning, Sichuan Agricultural University, Chengdu 611830, China; wanjiangjun@sicau.edu.cn (J.W.); 201608383@stu.sicau.edu.cn (Y.Z.); 2019225001@stu.sicau.edu.cn (K.Z.); lijia@stu.sicau.edu.cn (H.S.); 2020425034@stu.sicau.edu.cn (Z.W.); 201808259@stu.sicau.edu.cn (H.W.); limingjie@stu.sicau.edu.cn (M.L.); 41360@sicau.edu.cn (L.Z.); 41344@sicau.edu.cn (X.T.); yingcao@sicau.edu.cn (Y.C.); 201708620@sicau.edu.cn (L.T.); 2 State Key Laboratory of Geohazard Prevention and Geoenvironment Protection, Chengdu University of Technology, Chengdu 610059, China; 3 School of Economics, Sichuan Agricultural University, Chengdu 610101, China

**Keywords:** fitness, medical and health, POI

## Abstract

Background: The world faces vast health challenges, and urban residents living in high-density areas have even greater demand for healthy lifestyles. Methods: Based on the data of points of interest, a field survey, and an interview, we explored the healthy community-life circle in the downtown area of Chengdu, China from two perspectives: objective measurement and subjective perception of residents. We evaluated the coverage rate and convenience in accessing eight types of health service facilities within a 15-min walk using linear and logistics regression models to explore the degree of resident satisfaction with facilities and influencing factors. Results: Results showed significant differences in coverage rates between different districts. The overall convenience in accessing health service facilities decreased gradually from the city center to the outskirts. The social environment, the layout of health service facilities, and residents’ travel habits were related to health service facility satisfaction. Results also showed significant differences in various facilities’ accessibility satisfaction between objective measurement and residents’ perception measurement. Compared with subjective measurement, the objective measurements of accessibility for sports venues (objectively measured average minus perceived average: −1.310), sports zones (−0.740), and specialized hospitals (−1.081) were lower; those for community hospitals (0.095), clinics (1.025), and pharmacies (0.765) were higher; and facility accessibility measured by subjective perception had a more significant impact on health facility satisfaction. Pharmacies (OR: 1.932) and community hospitals (OR: 1.751) had the largest impact among the eight types of facilities. Conclusion: This study proposed to construct a healthy community-life circle with a category and hierarchy system.

## 1. Introduction

In the 21st century, the population is continuing to increase rapidly, and the speed of urbanization is accelerating. According to the United Nations Population Division statistics, the global population in 2019 reached 7.7 billion [1], an increase of 1.7 billion since 2000. The world faces huge health challenges, including health equity, obesity, and disease; unhealthy diet; lack of exercise; and other issues [2], forcing people to think repeatedly about preventive, healthy lifestyles. Since the outbreak of the COVID-19 epidemic, many countries have adopted measures to restrict residents’ travel and range of activities to prevent the spread of the virus [3], increasing the demand and willingness to improve health service facilities in the community and surrounding areas. According to the World Health Organization’s latest statistical report 2021, the Universal Health Coverage Service Index increased from a global average of 45 (100) in 2000 to 66 in 2017, indicating that more and more people can access high-quality health services without financial difficulties. However, as a special public event, the emergence of COVID-19 and policy control has posed major challenges to the planning and management of urban health service facilities.

Physical activity is always an effective means to maintain health, but studies have shown that global physical activity levels have not improved since 2001. In 2016, over a quarter (1.4 billion) of the world’s adult population was physically inactive, putting it at risk of noncommunicable diseases and premature death [4]. Lack of physical activity causes 5.3 million deaths every year globally, and the COVID-19 pandemic has further frustrated the slow-moving work of promoting sports activities of global residents [5,6]. Some scholars have pointed out that the government must promote physical activity as a basic human need beyond and independent of COVID-19 [4]. As residents have increasingly demanded physical exercise, they have also paid more attention to the surrounding medical service facilities. Accessibility and quality of nearby medical treatment have become critical criteria for residents to measure the community space and management level [7]. At the same time, achieving equitable access to health care is an essential embodiment of social equity [8]. In response to the COVID-19 epidemic, temporary hospitals were built to treat severe patients in many Chinese cities, which played a huge role in China’s rapid control of the spread of the epidemic. Thus, medical and health facilities play a decisive role in health emergencies. Therefore, rational layouts and availability of spaces for fitness activities and health care facilities are critical in the fight against various urban disasters and public health emergencies and have great significance for improving the health level of the world’s public [9].

The health of the community has become one of the most critical issues for governments in formulating public health and planning policies in metropolitan areas [10]. The World Health Organization was the first to put forward the concept of a “healthy community”, which extends the concept of health to the whole community. All community organizations can cooperate to improve the quality of life of all residents. To date, the research on healthy communities has focused only on evaluation index systems and standards of healthy communities; design and construction strategies for healthy communities; the participation of residents in the construction of healthy communities; and the governance model and management systems for healthy communities. The research on health-related service facilities has focused on their geographical distribution, but these models have largely reflected previous planning layouts. Although the needs of residents have been considered from the regional population scale, the rational layout of the life circle has seldom been made from the actual perception and evaluation of residents.

Following the concept of the healthy community, the idea of 15-min community-life circles was proposed. The Japanese government first proposed the concept of a “community residential area”, which refers to an area within a radius of 1000–2000 m, a 15-min walk from the residential center, where residents can obtain their daily needs [11]. The concept later developed into a “community-life circle”, introduced to China in the early 1990s. In recent years, cities such as Beijing, Shanghai, and Hangzhou have announced the establishment of 15-min community-life circles. The 15-min community-life circle is the space, centering on a person’s residence, in which they carry out various daily activities, including shopping, leisure, medical visits, education, employment, commuting, and other life-related services, which emphasizes walking as the transportation mode [12]. The new urbanism proposed in the 1990s also combined the elements of traditional housing design in walkable and mixed-use blocks instead of the typical low-density and curved-street layout of traditional suburbs. Research on the community-life circle has focused mainly on the configuration of public service facilities. The coverage rate [13], compliance rate [14], completeness and quality [15], and travel accessibility of various public service facilities on residential land in the community have been taken as indices [16,17,18,19]. Various public service facilities were analyzed using quantitative point of interest (POI) and geographic information system (GIS) approaches [20].

In studies on the optimal configuration of the 15-min community-life circles, community-life circles have usually been divided based on streets, administrative boundaries, and community buffer zones formed by people walking for 15 min. Most studies have used methods such as the standard deviation ellipse, the minimum convex polygon, and nuclear density analysis. However, these methods and standards for demarcating boundaries have some problems. They cannot reflect the actual 15 min community-life circle range of residents [21]. For community-life circle studies, the main focus has been on configuring comprehensive public services within community circles. Although some special studies on medical and health facilities and sports, leisure, and fitness activities have been conducted, the research focusing on healthy community-life circles is still relatively weak. Some of this research has been limited to the macro level, without considering the needs of different residents.

The fitness facilities are known as health promotion services and resources include green parks and squares, sports venues, and sports zones, which provide people with opportunities to exercise and improve their health. The most important resources for health protection are medical and health facilities. Primary health care is the first point of entry between patients and the health system. Thus, strengthening primary health care at the community level is key to achieving universal health coverage. Scholars have conducted relevant research on all of these facilities. Most research has focused on assessing the accessibility, availability, and fairness of parks and squares, fitness facilities, and medical facilities at all levels [22,23,24]. GIS tools and two-step floating catchment area methods are often used to analyze and improve evaluation methods continuously [24,25]. To understand the relationship between leisure and fitness spaces and health further, Zhang et al. explored the impact of parks and sports venues on people’s physical activities [26]. Furthermore, many scholars have compared differences in access to health-related resources among different groups and explored the impacts of differences in health; among these groups, the elderly and the poor have received special attention [27,28]. Previous studies have examined singular aspects of fitness facilities or medical and health facilities but have rarely combined the two to assess the impact on health. Some scholars have integrated health into the 15 min community-life circles and proposed to build a healthy community-life circle. On the one hand, aiming at the growing number of chronic diseases, urban planners need to add green space, open space, fitness facilities, and slow walking systems with high accessibility to promote residents’ physical activity and social interaction. On the other hand, aiming at the epidemic emergency, urban planners need to increase institutions and facilities related to epidemic emergencies to meet the need for prevention, isolation, treatment, and assistance during an epidemic [29]. The construction of healthy community-life circles can delineate the basis for the research scope and provide proposed path guidance for planning proposals. The most critical thing is that the healthy community-life circle connects health service facilities with people’s life circles. Therefore, healthy community-life circles were constructed that included medical and health facilities and fitness activity facilities as the basis of the current study.

Many scholars believe that accessibility is an effective means to evaluate the rationality of the layout of public service facilities. When accessibility is associated with walking, it is considered to be an indicator to measure the quality and operational effectiveness of a community. Walking accessibility is a critical factor among residents’ ability to benefit from facilities, resources, and services and is considered a measure of whether a community’s built environment encourages people to walk [30,31]. In recent years, the quality of the walking environment has gradually become an essential element of urban planning and design, and more and more studies have attempted to explore the relationship between, and influence the effect of the former on the latter for, characteristics and attributes of the neighborhood environment and walking. Walking has attracted more and more attention from scholars, mainly because it can affect people’s physical and mental health, promote the balanced development of urban and public service facilities, and improve community satisfaction [32,33]. As walking is an important means of transportation, walking accessibility has often been regarded by scholars as an important indicator to analyze the fairness of facility distribution in studies on evaluating the distribution of park squares, sports activity facilities, and medical and health facilities [34,35,36]. These studies have mainly used surveys, global positioning systems, GIS, and other methods to study the correlation between neighborhood environment and walking empirically, which provided a basis for studying neighborhood environments suitable for walking.

In addition to walking accessibility, perception evaluation also plays a key role in influencing community environmental satisfaction. The neighborhood satisfaction model constructed by Amerigo and Aragones in 1997 conceptualized the residential environment as objective and subjective attributes [37]. Some studies have found inconsistency between perceptual and objective measurement. In the existing literature, the objective evaluation of neighborhood environmental attributes has mainly relied on the GIS, whereas the subjective measurement method has usually involved collecting residents’ perceptual data for evaluation and analysis [38,39]. Furthermore, some studies have attempted to combine quantitative analysis based on objective data, such as GIS data, with qualitative analysis based on subjective/perceived data collected from residents for comparative and comprehensive analysis to explain the impact of neighborhood environmental attributes fully [40]. Although some studies have shown that subjective assessments of community environments are more important than objective assessments in explaining community environmental satisfaction [41], objective measurements have the advantage that they can be accessed through public datasets. They have the potential to guide urban planning better based on their specific and repeatable parameters. Thus, this study combined the objective measurement method of the GIS and the subjective measurement method of the questionnaire interview to explore the difference in terms of health service facilities’ walking accessibility between the perceived satisfaction of residents and objective measurement.

Thus, this study took the community-life circle of residents in the main urban area of Chengdu as its research focus to investigate the layout of community medical and health facilities and fitness facilities from the perspective of healthy urbanization development and explore the weak links in the city’s development. This study aimed to solve three problems: (1) from a macrospatial perspective, to construct an evaluation index system for healthy community-life circles in big cities and evaluate the strengths and weaknesses of these healthy community-life circles objectively; (2) from a micro perspective, to explore the difference between residents’ micro perception and objective measurement in the healthy community-life circle and analyze the key factors that affect the satisfaction of health service facilities; (3) to examine how to build healthy community-life circles reasonably and improve residents’ satisfaction with said healthy community-life circles. Our study offers the following potential contributions: exploration of the layout of healthy community-life circles of the metropolis and key influencing factors affecting health service facilities based on macrospatial analysis and the micro perspective of residents’ awareness and evaluations of the community; constructing a new theory of healthy community-life circles and putting forward corresponding optimization suggestions and policies on the basis of the theory of the original community-life circle in Chengdu. Furthermore, this study intended to use a novel analysis method: comparatively analyzing the perceived and objectively measured accessibility of health service facilities. Unlike traditional health facility studies that mainly focus on medical and health facilities, this study combined fitness facilities and medical and health facilities to analyze together to develop a new conceptual framework for a healthy community-life circle. This study provides a basis for optimizing healthy community-life circles in China and a reference for decision makers in urban planning.

## 2. Research Area and Data Resources

### 2.1. Study Area

**Figure 1:**
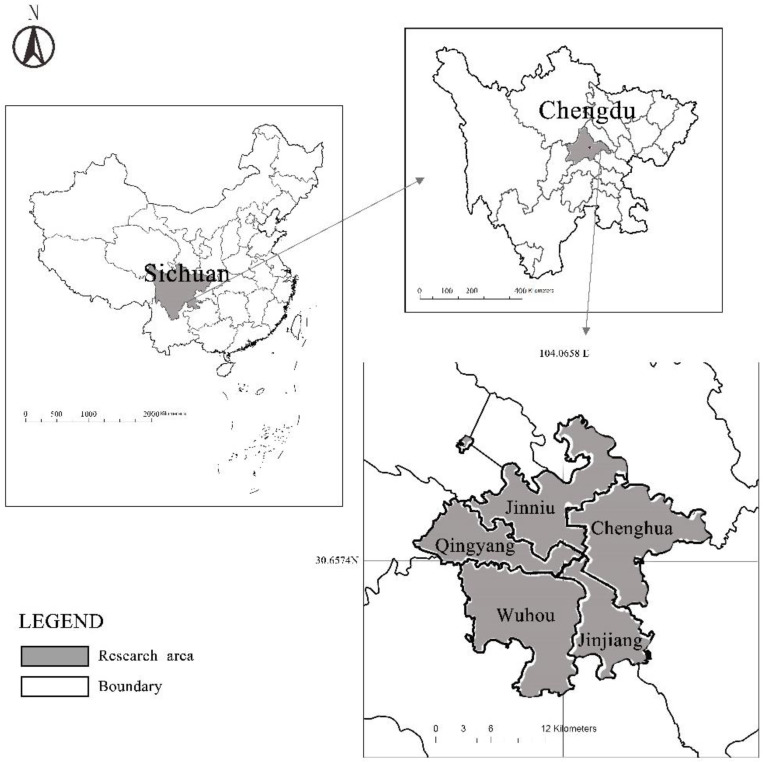
Location of the study area.

The research area in this study was the central city of Chengdu, Sichuan Province (104:04 E and 30:39 N) (Figure 1), which includes Jinjiang District, Qingyang District, Jinniu District, Wuhou District, and Chenghua District. According to official government statistics in 2020, the gross study area was approximately 424.06 square kilometers, holding a population of 4.232 million in 2019. According to the local government’s population data, the population density in the core urban areas was 10,300 people per square kilometer by the end of 2016. Chendu’s population is expected to be close to or exceed the critical value for pressure on natural resources by 2035.

High-quality, harmonious, and livable communities are currently the focus for improving the quality of the living environment in Chengdu. In December 2018, Chengdu issued the “Working Plan for Creating a New Community Commercial Area and Building a Community Quality-of-life Service Circle (2018–2022)”. It proposed promoting the construction of a 15 min community-life circle vigorously by taking the community as the central point to allocate services for the population scientifically and reasonably using a set radius to ensure that basic public services could be reached within 15 min of travel on foot or by bicycle. Therefore, downtown Chengdu was taken as the case study herein.

### 2.2. Data Sources

This study’s data mainly came from spatial POI data and field survey data. The POI data consisted of specific point data of spatial entities closely related to daily lives with accurate geographic and attribute information including longitude, latitude, name, address, type, and label [42,43]. They provided accurate locations and detailed categorical information for commercial businesses, services, and public places and had the advantages of being abundant and free to access [44]. Using the Python programming language, websites were crawled by looping through the URLs to collect road network and POI data for the Jinjiang, Qingyang, Jinniu, Wuhou, and Chenghua Districts of Chengdu in 2019. In the collected POI data, after screening and checking of locations and attributes, 3658 POI data in the study area were selected and used as the starting points of residents’ daily travel to measure the health service facilities within 15 min community circles, which was one of the bases for delimiting healthy community-life circles. Field research data came from the issuance of questionnaires and in-depth interviews with individuals. Finally, 371 valid questionnaires were obtained. Therefore, the data of this study were sourced from community POI and survey data.

## 3. Methods

**Figure 2:**
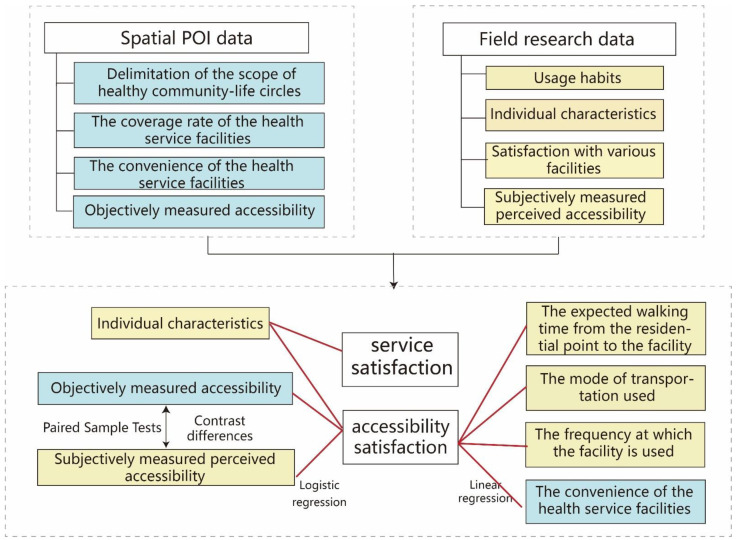
Research framework.

To evaluate healthy community-life circles, the Delphi method was used at the macro level to determine the weights of various health service facilities. The UNA tool was also adopted to delineate the boundary of each 15 min healthy community-life circle, calculate the coverage and convenience of each urban facility, and conduct comparative analyses between them. Questionnaires were distributed on site, and in-depth interviews with individuals were conducted to analyze resident satisfaction with facilities and influential factors. The data were analyzed in SPSS25. The test was conducted to establish the reliability and validity of the questionnaire to verify the scientific rigor of the research. This study thoroughly analyzed the influence mechanisms of objective measurement (coverage rate and convenience) and subjective, perceived satisfaction of residents using linear regression and logistic regression models (Figure 2).

### 3.1. Classification and Model Weighting of Health Service Facilities

Existing studies have shown that the use of parks, squares, and sports facilities exerts a positive influence on the improvement of mental and physical health and can also reduce the risk of anxiety and a variety of chronic diseases, whereas medical and health facilities are the most important safeguard for maintaining the health of the population [45,46,47]. Therefore, two broad categories were selected for evaluating the service facilities within healthy community-life circles: medical and health facilities and fitness facilities. The types of facilities included in these broad categories were determined by referring to the classification of medical and fitness facilities in previous studies [13,48]. Medical and health facilities were divided into five categories: general hospitals, specialized hospitals, community hospitals, clinics, and pharmacies. Strictly speaking, general hospitals and specialized hospitals are outside the scope of facilities in the healthy community living circle. However, Chinese residents have low trust in lower-level medical facilities, so they choose to go to large hospitals to treat even minor illnesses. Therefore, general hospitals and specialized hospitals were included in the facilities to be evaluated in this study. Fitness facilities were divided into three categories: parks and squares, sports venues, and sports zones. This study employed a classic Delphi method to ensure scientific and subjective weighting. As a long-term forecasting method, the Delphi method, which makes recommendations based on experts’ opinions, has been widely used for auxiliary decision-making [49]. We sent a letter to 20 experts introducing the study and the Delphi process and inviting them to participate in this phase, which was completed between August and October 2020. The experts were required to score the eight facilities in Table 1, ranging from “very important” to “not important”, from which a facility grade average was calculated. The order of importance of these health services was used to obtain the weights for the various analyses (Table 1).

### 3.2. Delimitation of the Scope of Healthy Community-Life Circles

Chengdu Municipal Government aims to optimize the spatial layout of the city’s medical and health resources and improve the accessibility of medical and health services by compiling the “Chengdu City Medical and Health Resources Layout Plan (2017–2035)” and proposing the construction of 15 min healthy circles that incorporate at least one community health service center within a 15 min walking distance (800–1000 m) for residents to carry out diagnosis and treatment measures. In this study, a healthy community-life circle was defined as integrating health into the 15 min community-life circle. In such a circle, within walking distance from home, various health service facilities can meet residents’ daily health needs, such as fitness exercises and medical services, and have health-promotion effects and the ability to respond to public health emergencies.

Previous studies have usually employed a certain straight-line distance as the scope of residents’ activities to delimit their community-life circles, ignoring factors such as buildings, roads, and topography. For the purposes of reflecting the scope of people’s community-life circles more truthfully, this study used UNA in Arc-GIS to construct a city network dataset for network data analysis. The selected community POIs were used as the starting points of residents’ daily travel, taking walking as the travel mode, streets as network links, and intersections of the streets as odes of the network to create the network dataset. In the “SHANGHAI PLANNING GUIDANCE OF 15-MINUTE COMMUNITY-LIFE CIRCLES” issued by the Shanghai Municipal Government, the 15 min walking distance is 800–1000 m, and people’s normal walking speed is between 0.75–1.2 m per second [50]. Given the speed differences between different genders and age groups, this study adopted an average speed of 1.0 m/s as the residents’ walking speed. To build a new service area, the walking mode was chosen as impedance, the interruption value was 15 min, and the corresponding surface generated was the actual walking range of the residents in 15 min. This mode was used within the constructed network to generate a surface that represented residents’ actual walking range in 15 min. This method better reflected the distance people walk in 15 min and more scientifically delimited the 15 min community-life circle. For larger communities, given the greater differences in the coverage area of a 15 min walk, these areas were divided into several smaller surfaces, and the corresponding centroid was generated as the starting point [51].

### 3.3. Coverage Rate and Convenience Evaluation

This study used Arc-GIS to calculate the coverage rate and convenience in accessing medical and health facilities and fitness facilities. Based on the selected POI data, the coverage rate of the health service facilities in the community-life circles was measured. If a particular type of facility was reachable within a 15 min walk, the living circle was said to be covered. After calculating the coverage rate of each facility, the overall coverage rate of health service facilities in each community-life circle was determined:

(1) $$C_{i,j,s}=\{\begin{array}{c} 1,\exists F_{j,}\in N_{1}\left(Community_{i,s}\right)\\ 0,others \end{array}$$

(2) $${\mathrm{CR}}_{i,j}=\left(\sum_{s=1}^{m_{i}}C_{i,j,s}/m\right)/m_{i}$$

(3) $$F_{i}=\overset{n}{\underset{j=1}{\sum }}F_{\mathrm{ij}}\times W_{j}$$

where C_is_ represents community s in the urban area i; C_ijs_ represents whether there is a health service facility F_j_ within the community-life circles (if it exists, this means it is covered); CR_ij_ represents the coverage rate of the health service facility F_j_ in the ith urban area, showing the coverage level of different areas; m_i_ is the number of living circles in the urban area i; F_i_ is the overall coverage rate of the health-related facilities in the ith community-life circle; F_ij_ is the coverage rate of category j facilities in the ith community-life circle; and W_j_ is the weight of the j facilities.

The coverage rate reflected only the overall spatial distribution of the various facilities. It was also necessary to define the convenience of residents in each healthy community-life circle in accessing various health service facilities. Arc-GIS was used to calculate the number of each kind of facility that was accessible within each community-life circle. The convenience of accessing the health service facilities in each community-life circle was graded according to this number. The grading of convenience followed the natural discontinuous grading method, which divides the elements into multiple classes. For these classes, the boundary was set at the position where the differences in the data values were relatively large, keeping similar group values appropriately grouped while maximizing the difference between each category. On this basis, the convenience in accessing the various health facilities was divided into eight levels, and the convenience in accessing the various health service facilities in the healthy community-life circles was evaluated.

### 3.4. Questionnaire Design and Distribution

The questionnaire mainly investigated residents’ usage habits and satisfaction with various facilities, the distribution of various facilities near their places of residence, their suggestions for the improvement of various facilities, and protective measures they had taken following the outbreak of the COVID-19 epidemic. The questionnaire was based on four dimensions: frequency of facility use, walking time from residence to the facility, the ideal walking time between residence and facility, and satisfaction evaluation. Satisfaction included two aspects: residents’ satisfaction with accessibility of health service facilities and service satisfaction. In the questionnaire, each respondent evaluated their satisfaction with each health service facility’s accessibility and service (general hospitals, specialized hospitals, community hospitals, clinics, pharmacies, sports venues, sports zones, parks, and squares) closest to home. A five-level Likert scale was adopted to provide satisfaction indexes. This study assigned values as follows: 5 points—very satisfied, 4 points—relatively satisfied, 3 points—generally satisfied, 2 points—relatively dissatisfied, and 1 point—very dissatisfied. The walking time was divided into levels of “less than 5 min”, “5–10 min”, “11–15 min”, “15 min or more” and “unclear” for the purpose of analyzing the relationship between walking time and satisfaction.

Considering the impact of different individuals on satisfaction, we also classified individual factors, including gender, age, education, job, annual income, type of dwelling, and other variables (Table 2). The “type of dwelling” option was based on the respondents’ answer to the question “Which one of the following does your residential area belong to?” The classification standard of residential type referred to previous studies and the actual situation of Chengdu. The type of dwelling was divided into four categories: (1) low-end housing types, including shanty towns; old, unrenovated city housing; and agricultural residential houses; (2) mid-range houses, including ordinary commercial houses; (3) high-end houses, including villas and high-end commercial houses; and (4) units and school dormitories. During the survey, randomly sampled interviews were conducted with residents in the five urban areas of Chengdu in different time spans selected between 9:00 and 22:00 to obtain data for different periods. First, a presurvey was conducted, during which 63 questionnaires were issued. After all valid presurvey questionnaires were collected, reliability and validity tests (see details below) were conducted on the data. After deleting a single invalid question, the final questionnaire was formed, and the survey was carried out.

(1) Reliability Test

Reliability refers to the degree of consistency and stability of the data obtained from multiple responses to the questionnaire. Values range from 0 to 1. The larger the value, the higher the reliability. To take into account the repeated tests, Cronbach’s reliability coefficient method was adopted (abbreviated as a). The mathematical formula of Cronbach’s alpha reliability coefficient is:

(4) $$a=\left(k/\left(k-1\right)\right)\left(1-\left(\overset{k}{\underset{i=1}{\sum }}\sigma_{i}^{2}\right)/\sigma_{T}^{2}\right)$$

where K represents the total number of items, σ_i_^2^ represents the in-question variance of the score of the ith question, and σ_T_^2^ represents the variance of the total score. The 40 variables were statistically analyzed, and the output showed that the Cronbach’s alpha reliability coefficient was 0.885, indicating that the reliability of the data was excellent, so further analyses could be performed.

(2) Validity Test

Reliability examines the consistency across all items in the scale, while validity specifically examines the consistency of each individual item, that is, whether each item plays an important role in the scale [52]. To test the suitability of the data, it was necessary to perform the KMO (Kaiser–Meyer–Olkin) and Bartlett sphere tests on the samples. The KMO index was 0.861 (Table 3), which was close to 1, indicating that the data was suitable for factor analysis.

### 3.5. Data Processing

The study evaluated residents’ accessibility and service satisfaction for fitness and medical and health facilities by calculating the average and standard deviation to analyze the basic situation of residents’ accessibility satisfaction evaluation.

The differences among the interview subjects had specific impacts on their satisfaction scores, among which differences in gender, age, education level, occupational status, annual income, and housing type were considered. We constructed an ordered multiclass logistic regression model to analyze the impacts of the differences in the subjects on satisfaction.

We adopted a classical linear regression analysis, taking satisfaction with the eight types of facilities as the dependent variables. The frequency at which the facility was used, the mode of transportation used to get there (What is your most common mode of transportation to the following locations?), the perceived walking time from the residential point to the facility, the expected walking time from the residential point to the facility, and convenience (according to the place of residence filled in the questionnaire, convenience was evaluated in a city network dataset) were used as independent variables to explore the factors influencing residents’ satisfaction scores. Because the transportation mode used by residents was a categorical variable, it was analyzed with a chi-square test. Chi-square is a nonparametric hypothesis-based method used to calculate the degree of fit between actual observation values and the theoretical inferred values of two or more samples. The calculation formula is as follows:

(5) $$x^{2}=\sum {\left(A-T\right)}^{2}/T$$

where A is the actual observation value and T is the theoretical inferred value. By using SPSS, chi-square test results for the eight health service facilities were obtained.

Paired-sample T-tests were used to compare and analyze the differences between the residents’ perceived value and the actual value of the distance between their residences and the facilities. Residents’ perceptions of the walking time from their residential areas to the facilities were assessed by the subjects’ responses to the question “from home, the time to walk to the nearest place below is”. The answers included “less than 5 min”, “5–10 min”, “11–15 min”, “16–20 min”, “more than 20 min”, and “unclear”. The actual distances between their residence and the facilities were based on the addresses filled in by the respondents. A network dataset was created in Arc-GIS to generate the corresponding range and calculate the actual walking time of 5, 10, 15, or 20 min from their residence to the nearest facility. The samples with unclear answers selected in the questionnaire were deleted, and a paired sample *t*-test was performed on the remaining actual and perceived values. Simultaneously, we constructed a logistic regression model for eight health service facilities to explore the relationship between objective and perceived measures of accessibility of and satisfaction with health service facilities.

## 4. Results

### 4.1. Evaluation of the Healthy Community-Life Circles

#### 4.1.1. Evaluation of Spatial Differences in the Coverage Rate of Health Service Facilities

**Figure 3:**
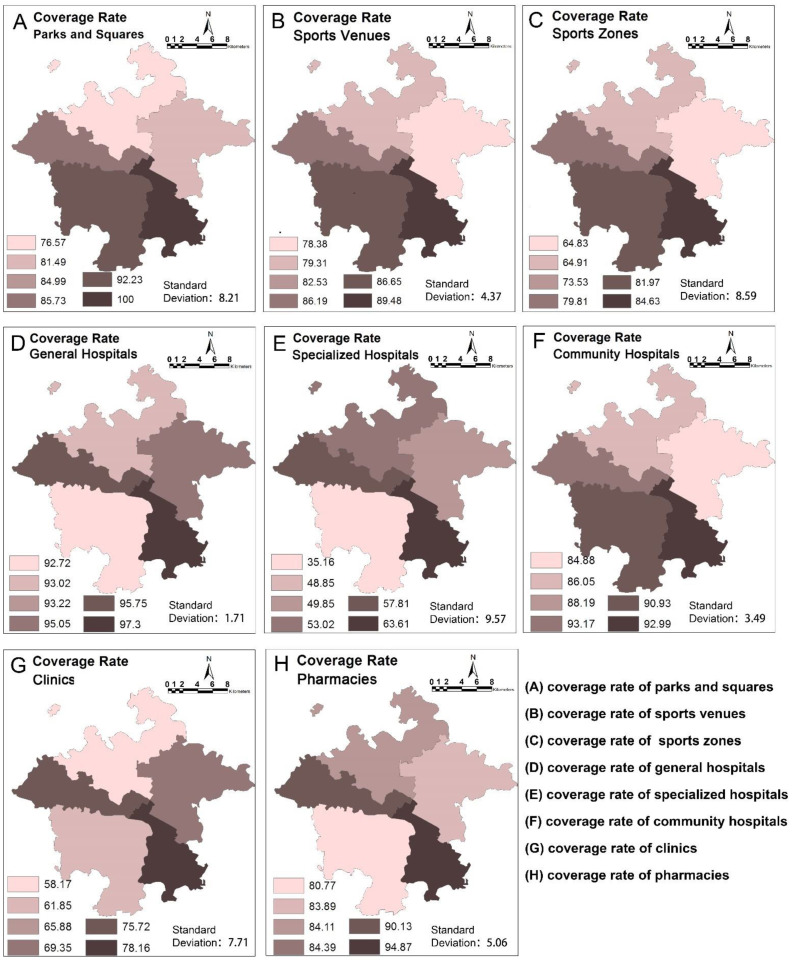
Coverage rates of health facilities within the 15 min community-life circles in the main urban area of Chengdu.

The average coverage rates of the various health-related facilities in the main Chengdu urban area were general hospitals (93.22%) > community hospitals (88.19%) > parks and squares (84.99%) > pharmacies (84.39%) > sports venues (82.53%) > sports zones (73.53%) > clinics (65.88%) > specialized hospitals (48.85%). The corresponding standard deviations were specialized hospitals > sports zones > park and squares > clinics > pharmacies > sports venues > community hospitals > general hospitals. The greater the standard deviation, the greater the difference in the average coverage rate of the facility in each urban area, and the more uneven the overall distribution of the facility in downtown Chengdu. Figure 3 and the sizes of the standard deviations show that the two facilities with the most pronounced differences between urban areas were the specialized hospitals and sports zones. These two types of facilities had low coverage rates in each urban area as a whole.

#### 4.1.2. Evaluation of the Spatial Characteristics of the Degree of Convenience in Accessing Health Service Facilities

**Figure 4:**
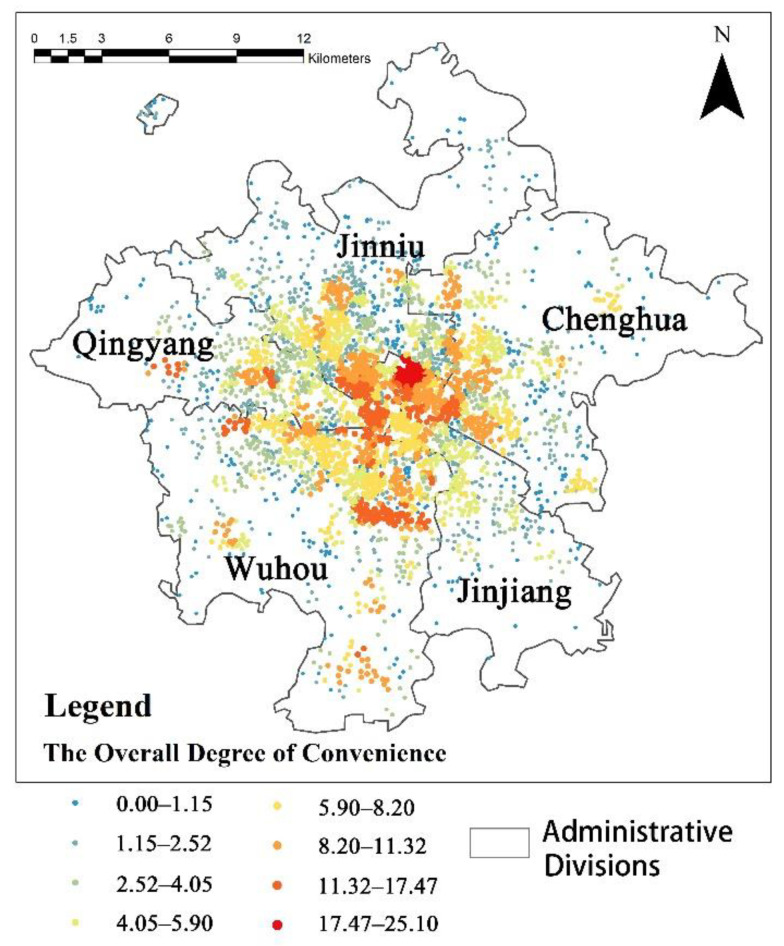
Analysis of the overall degree of convenience and comprehensive assessment of eight types of facilities.

**Figure 5:**
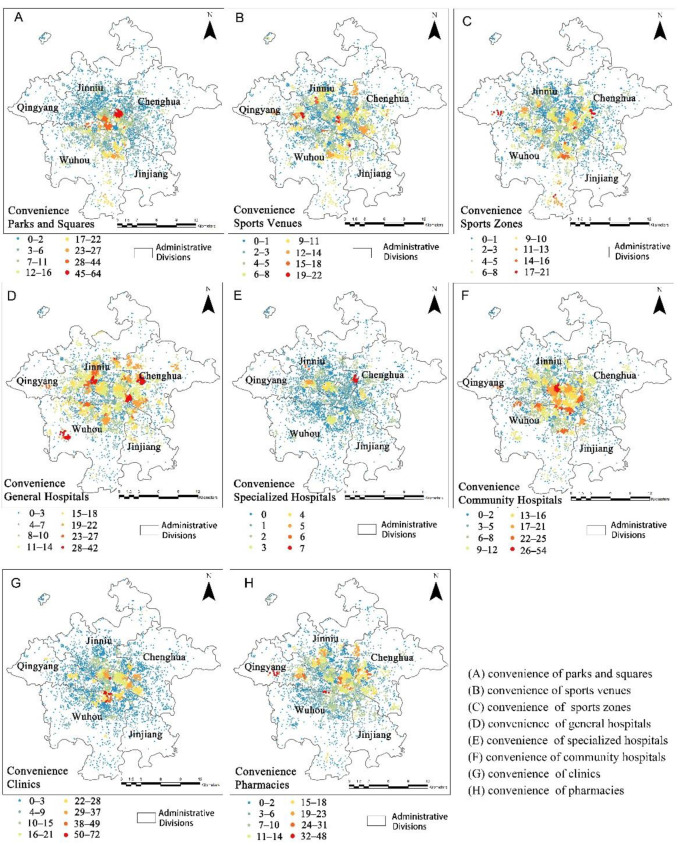
The degree of convenience in accessing various facilities.

From the results presented in Figure 4, we drew the following inferences: the overall convenience index of the five urban areas of Chengdu gradually decreased from the city center. Figure 5 shows that the park and square facilities in the Qingyang District offered a high level of convenience. At the highest level, 64 parks and squares were accessible, and the areas with highly convenient parks and squares were concentrated. Most other urban areas had lower convenience scores for parks and squares. The gap in convenience scores for sports venues in the different urban areas was not significant. The scores were distributed more uniformly within healthy community-life circles. The distribution of sports zones did not follow the trend of decreasing from the city center to the outside. The convenience scores of general hospitals and community hospitals were generally higher than those of other facilities, but the convenience scores of general hospitals in urban centers were not high. The convenience scores of specialized hospitals were the lowest of all of the facilities. The areas with high convenience scores for clinics and pharmacies were mainly concentrated in the central urban area, and most of the central area had high convenience scores for pharmacies.

### 4.2. Evaluation of Resident Satisfaction and Analysis of Influencing Factors

#### 4.2.1. Evaluation of Resident Satisfaction Results

According to Table 4, the overall satisfaction with accessibility (3.20) was slightly higher than the satisfaction with service (3.12), which indicates that residents were relatively satisfied with the accessibility and services offered by these eight types of facilities. No significant difference was found in the average values of accessibility and service satisfaction of residents for the same health service facilities, indicating that facilities’ service quality and accessibility may jointly affect the evaluation of residents’ satisfaction. Residents were more satisfied with the accessibility and service of pharmacies and park squares. Satisfaction with specialized hospitals, sports venues, and sports zones was low, and the standard deviation was significant, indicating that residents’ satisfaction with these three facilities was significantly different.

#### 4.2.2. Residents’ Satisfaction Was Related to Their Attributes and Usage Habits

Table 5 and Table 6 show a significant positive correlation among age, occupational type, and accessibility and service satisfaction of health service facilities. With increasing age, residents’ satisfaction with the walkability of health service facilities and service quality gradually increased. Residents with permanent jobs generally had higher satisfaction than those without permanent employment. Furthermore, compared with residents with annual incomes of more than 100,000 yuan, residents with annual incomes of less than 50,000 yuan rated the accessibility and service quality of park squares and pharmacies more positively. Residents of upscale neighborhoods reported poorer satisfaction with the accessibility of pharmacies and clinics than those living in dormitories.

Table 7 shows that only the frequency of residents using community hospitals positively correlated with accessibility satisfaction. In contrast, the frequency at which other facilities were used had no significant impact on their accessibility satisfaction. No linear correlation was found between the degree of convenience and the degree of satisfaction with the accessibility of various facilities. In the analysis of the relationship between the accessibility satisfaction with various facilities and the mode of transportation used by residents, we found that walking mode had a positive effect on improving the accessibility satisfaction of parks and squares, sports venues, sports zones, community hospitals, clinics, and pharmacies. There was no significant correlation between walking and accessibility satisfaction with general hospitals and specialized hospitals (*p* > 0.1). However, using nonmotor vehicles to travel to specialized hospitals could significantly improve accessibility satisfaction of specialized hospitals. Therefore, we can infer that walking was not the best choice for either facility in the 15 min healthy community-life circle. The preference to use nonmotorized vehicles or public transportation also positively affected satisfaction with the accessibility of sports venues, sports zones, community hospitals, and clinics. By analyzing the relationship between the expected walking time and the satisfaction of eight types of health service facilities, we found that the shorter the expected walking time was, the higher the satisfaction evaluation of residents was.

### 4.3. Resident Perception and Objective Measurement Analysis for Health Service Facilities

#### 4.3.1. Analysis of Residents’ Perceptions of Distance between Residential Sites and Facilities

Table 8 shows that residents’ perception of various health service facilities around their living places was inaccurate. First, in the paired sample correlation test, except for sports zones (*p* < 0.05), the *p*-values of all of the other facilities were >0.05, indicating a significant correlation between the objectively measured distance to sports zones and people’s perception of the distance. No significant correlation was found between the objectively measured and perceived distances for the other seven facilities. In the paired sample tests, the *p*-values for the sports venues, sports zones, specialized hospitals, community hospitals, clinics, and pharmacies were less than 0.05, indicating significant differences between the perceived values and the objectively measured values, and no significant differences were found between the perceived and objectively measured values for parks and squares and general hospitals. The average differences in the objectively measured and perceived values for sports venues, sports zones, and specialized hospitals were negative, indicating that the objectively measured values were significantly lower than the perceived values. The average differences in the objectively measured and perceived values for community hospitals, clinics, and pharmacies were positive, indicating that the objectively measured values were significantly higher than the perceived values.

#### 4.3.2. Relationship between Objective and Perceived Measures of Accessibility of and Accessibility Satisfaction with Health Service Facilities

We constructed an ordered logistic regression model for eight health service facilities. Three facilities (sports venues, general hospitals, and clinics) had *p*-values under parallelism tests of less than 0.05, indicating that the grade spacing of dependent variables was not equal. Disordered logistic regression model analysis was required (Table 9). The accessibility satisfaction with all health service facilities in the table was significantly correlated with perceived accessibility. In contrast, no significant correlation was found with objective accessibility measures (Table 10), which further proves the importance of residents’ perception of accessibility satisfaction. By comparing the OR value of perceptual measurement in each model, we analyzed the impact of residents’ perceived accessibility between different health service facilities and its implications. Pharmacies and community hospitals had the most significant effect of the eight types of facilities. When the perceived accessibility level of pharmacies and community hospitals increased by 1 unit, the probability of improving the accessibility satisfaction level of residents was 93.2% and 75.1%, respectively. These were followed by sports zones, parks and squares, and specialized hospitals.

## 5. Discussion

### 5.1. Nonuniform Spatial Distribution in Health Service Facilities

Although the number of health service facilities was relatively evenly distributed across various urban areas, the development of various facilities was disproportionate, and the overall convenience in accessing facilities near the city center was significantly higher than that in other areas.

Regarding coverage and convenience of comprehensive sports facilities, the distribution of sports zones in the healthy life circle was worse than that of the other two facilities, and satisfaction with their service (2.87) and accessibility (2.99) was also relatively low. Sports zones’ construction time and cost are lower than those of other facilities, but the current situation is still far from satisfactory. Do residents prefer to use other fitness facilities? Are planners too focused on parks and squares, neglecting sports zones? Is there another reason for this phenomenon? Further discussion is needed. The park and square facilities in Qingyang District were slightly more convenient than those in other urban areas, mainly because all kinds of large and small parks and squares in downtown Chengdu, such as Huanhuaxi Wetland Park, People’s Park, and Cultural Park, were concentrated in the central area of Qingyang District. Most of the parks were close enough for residents to use conveniently. Although other urban areas had large parks, the geographical location of these large parks was far away from residential areas and could not radiate to more residential sites. Therefore, small parks and squares were important to improve the construction of healthy community-life circles. Studies have shown that people prefer to use the boundary of the park space rather than entering the park, and that if a park or square’s shape is more complex, the usage rate of the residents is higher [53]. It is better to build a strip or ribbon park according to the river and terrain than to build a park square, as the former can cover more residential areas and improve the usage rate of residents. More complex or varied shapes should be designed in the planning of new parks and squares.

After establishing the 15 min healthy community circle in Chengdu, as shown in the study results, the convenience and coverage of community hospitals emphasized by the healthy community circle were above the medium level. However, the distribution of general hospitals and specialized hospitals was far less even than that of community hospitals, and the coverage of specialized hospitals and clinics was also lower, which may be related to the incomplete coverage of the population in specialized hospitals and clinics being able to provide only basic health services. Furthermore, most respondents (53%) said they barely used specialized hospitals and clinics. On the one hand, the distribution of the two types of facilities was uneven. On the other hand, the number of specialized hospitals was smaller, and the targeted audience was relatively single. The clinic use group is mainly a low-consumption crowd. People with medical insurance are less likely to use clinics. Specialized hospitals and clinics are all private hospitals, and urban residents prefer to use public hospitals with higher quality and usage levels [54]. Chengdu carried out graded diagnosis and treatment measures in the existing health circle. Clinics and community health centers played an important role in the initial stage of graded diagnosis and treatment. Although during the COVID-19 pandemic in China, higher-level general hospitals have taken on most of the treatment work for COVID-19 patients and received more attention and investment, clinics have played a considerable role in late-stage COVID-19 screening, vaccinations, and residents’ daily hospital visits. Planners should consider how clinics can play an important role in the healthy life circle.

### 5.2. Residents’ Behavior Characteristics Affect Their Satisfaction with Various Health Facilities

With the growth of age, the walking range of middle-aged and elderly residents becomes smaller, and their walking accessibility satisfaction with health service facilities may decrease. However, our results showed the opposite. This does not indicate that the activity ability of middle-aged and elderly residents was enhanced but may be related to the mental and vision changes brought by age. At the same time, one reason to consider is that young residents spend more time working and commuting than middle-aged and elderly residents. Less time at their disposal may lead to higher requirements for the accessibility of health facilities.

Residents’ estimated time needed for travel may affect residents’ medical choices [55]. Our results showed a positive correlation between the expected walking time required from the residential site to a hospital and the degree of satisfaction with the accessibility of said hospital. This implies that residents do not want to live close to hospitals, which may be because most residents use hospitals less frequently, while hospitals bring more negative effects (e.g., infectious diseases, pedestrian traffic, and security concerns) to residents. Therefore, some measures should be taken around general hospitals to reduce this phenomenon, such as using commercial entertainment facilities or green space to separate residential areas from general hospitals.

Although, on average, residents were relatively satisfied with the levels of service at the studied facilities, the interviewees still pointed out the shortcomings of various health service facilities. Nearly half of the interviewees (45%) thought that the number of sports venues was insufficient. Some interviewees also pointed out that the stadiums near their homes had short opening times, old facilities, poor environments, sanitation concerns, and high fees and had been closed since the COVID-19 epidemic outbreak, among other problems due to insufficient policy support, investment, and imperfect management by Chengdu’s stadiums [56]. The long waiting time for medical treatment was the issue most in need of improvement in general hospitals. The main problems for community hospitals and clinics were the limited medical functions available and the low level of medical care. Talen et al. found that the medical technology level of hospital facilities was the primary consideration for residents when choosing hospital treatment [57,58]. This finding was supported in the present study. Most interviewees preferred to select small medical and health facilities. However, given that the existing medical standards of community hospitals and clinics were unable to meet the medical needs of the residents, they were obliged to choose higher-level medical and health service facilities, such as general hospitals [59]. The supply and demand of medical facilities are imbalanced [60]. This study also found that more residents chose higher-level medical institutions (27.2%) than lower-level medical institutions (18.3%). The main reasons for choosing low-level medical facilities were affordability, residents’ fear of contracting COVID-19 in a health care environment, and residents’ delay or avoidance of medical treatment [1]. Some interviewees mentioned that private medical services (clinics and specialized hospitals) were not as good as public medical services or that they were not clear about whether their medical insurance could be used in private hospitals. Thus, they preferred to choose public medical and health facilities [61].

By analyzing the relationship between residents’ daily use of transportation and accessibility satisfaction of various health service facilities, we found that residents’ preferences for different health service facilities were different. For parks, squares, and pharmacies, only walking had a significant positive impact on improving accessibility satisfaction. However, for general hospitals, specialized hospitals, sports venues, sports zones, and other facilities, nonwalking transportation could also improve accessibility satisfaction. Thus, we believe that residents have different physical endurance when they go to various health service facilities and have “optimal” or “threshold” walking time. Given that general hospitals, for example, can provide higher-quality nursing services, and many interviewees may accompany their family members when they go to general hospitals, residents are more willing to pay higher transportation costs [54]. Thus, even within a 15 min healthy community-life circle, accessibility requirements may vary for different health service facilities. Only building walkable community-life circles may not be enough to meet the health needs of residents. Thus, the construction of healthy community-life circles should not be limited to 15 min healthy community-life circles. A model could be considered on the basis of the Chengdu healthy circle, that is, to build a 15 min walking healthy community-life circle to guarantee the basic healthy life of residents and a 15 min traffic accessible healthy community-life circle to meet all the healthy life needs of residents. However, promoting and improving healthy community-life circles still requires related research and actual verification in the future. This conclusion also has some generalized significance for other similar cities in the world.

### 5.3. Residents’ Perceptions of Health Services Accessibility Are the Key Factors Influencing the Degree of Satisfaction

Except for parks, squares, and general hospitals, the objectively measured accessibility of health service facilities was significantly different from the accessibility perceived by residents. This was consistent with the results of other studies [23,62]. This time, we adopted the perception of people living for a long time and reliance on experience rather than instantaneous time perception. Therefore, people’s daily habits, experiences, activities, or health degrees impacted their perception of health service facilities [63]. Furthermore, the residents’ perception of the physical environment was inseparable from the social environment [64]. The current social environment does not pay much attention to health. One reason why is that residents pay less attention to health facilities and services. Residents perceived fewer sports venues, sports zones, and specialized hospitals within a 15 min walk than the objectively measured number, and the numbers of community hospitals, clinics, and pharmacies were perceived to be greater than the objectively measured numbers. Combined with our field research, this study suggests that this perception may be related to the fact that some sports zones and sports venues are not open and do not attract residents’ attention. Thus, managers must strive to improve and maintain the environmental aesthetics of parks, squares, and small sports zones in the community to attract more people and enhance residents’ perceptions of them. At the same time, the health service department can promote physical activity through educational content and other means to improve the perception and health level of the population [65].

Our study further proved the importance of residents’ perception for accessibility satisfaction. Residents’ perceived travel time had a significant impact on their accessibility satisfaction with various facilities. The closer to their homes facilities were perceived to be, the more satisfied residents were with the accessibility of these facilities, and the objectively measured accessibility had no significant correlation. At present, studies have found that residents’ perception of the environment is more important than the objective reality. Of course, the lack of connection between objective measurement and accessibility satisfaction may be caused by some unstudied characteristics affecting residents’ perception in ways related to their satisfaction [40]. In future studies, it will be important to understand how changes in the neighborhood lead to neighboring health services being perceived by residents as adjacent. Although building more health service facilities would improve residents’ perceived accessibility and accessibility satisfaction, it is obvious that building facilities is insufficient for making qualified, healthy community-life circles.

### 5.4. Implications

The study results provided new evidence for constructing healthy community-life circles in China and have certain reference significance for the construction of similar cities and communities around the world. We believe that this study has contributed to the existing literature from two perspectives. First, unlike traditional health facility studies that mainly focus on medical and health facilities, the present study combined fitness facilities and medical and health facilities to analyze together, optimized the Chengdu healthy community-life circle, proposed building a healthy community-life circle with categories and hierarchical systems, and developed a new conceptual framework for a healthy community-life circles. Furthermore, a novel analysis method was adopted. Comparatively analyzing the perceived and objectively measured accessibility of health service facilities and emphasizing the importance of people’s perception of health service facilities are important concerns for policymakers but tend to be overlooked in many studies.

The results of this study also had several important policy implications. First, the government plays a leading role in the spatial configuration, planning, and construction of medical and health facilities and health service facilities, such as parks and squares; thus, the government should reoptimize the layout according to people’s needs [66]. The transportation system should be supplemented and improved to make up for the current low usage rates of pedestrian transportation to general hospitals, large sports venues, sports zones, specialized hospitals, community hospitals, and clinics. Furthermore, given the difficulty of building large-scale sports facilities in existing cities, the support of the private sector by the state and society is crucial for the development of fitness facilities [35], and improvements to the opening hours and accessibility of existing facilities are needed. For example, extending the business hours of public gymnasiums and opening school gymnasiums and playgrounds during nonclass time to the public are effective and feasible options [67]. Venues that offer activities suitable for different age groups could be added. As one interviewee mentioned, “I think several adjacent communities can jointly organize a fitness facility or gym, and the activities and content of the facilities could be specific for children, young people, women, and the elderly. Their physical needs and living habits are different, and some facilities are not suitable for the elderly or young people”. For existing fitness facilities, such as parks, squares, and other fitness facilities, attention should be paid to the maintenance of their environment and their content. When we surveyed satisfaction with parks, an interviewee said, “Now, the park is too noisy and iteratively updated so quickly. Many of the memories and feelings of youth that once filled the park are gone. Now, it is sometimes strange to go to the park I used to go to because it lacks some cultural things”. Therefore, the quality of parks and squares affects their attractiveness to people, which is paramount. The government also ought to strengthen its support for the construction of small hospitals, especially community hospitals and clinics, and improve their medical level and coverage to promote better medical resource allocation [66]. Standardized policies or rewards should also be adopted to improve the service level of private medical service facilities.

Second, in addition to building and opening up new facilities, alternative or low-cost construction between facilities is a method worth considering. For example, health service facilities with similar functions can replace one another. Planning and maintaining appropriate pedestrian infrastructure improves residents’ perception of accessibility. Furthermore, when residents go to medical and health facilities urgently, the time and distance are the most important. However, distance and time may be less important for residents using sports facilities than their service and quality. Therefore, improvements should be made according to the different characteristics of different health service facilities.

Finally, policymakers should promote the intersection between neighborhood design and health and encourage the participation of citizens with different socioeconomic attributes in the planning and construction of healthy community-life circles to improve understanding and consider the diverse health needs of residents in the neighborhood environment development process.

## 6. Conclusions

The sudden large public health incident of the COVID-19 pandemic has made people more aware of the importance of healthy community-life circles. We obtained POI data and used GIS tools to evaluate the medical and health facilities and fitness facilities in the main urban areas of Chengdu. Using a questionnaire survey, we compared the accessibility of health service facilities as actually measured and residents’ perception of accessibility and analyzed the difference in the impact of each on facility satisfaction. The results showed that the social environment, the service level and layout of facilities, and residents’ travel habits were related to facility satisfaction. Residents could not accurately perceive the surrounding health service facilities. Objectively measured walkability did not significantly affect the satisfaction of the facilities. Only residents’ perception of low walking time between the residence and the facility positively impacted satisfaction. These factors can reduce residents’ negative perceptions by optimizing facility layout and replacing low-cost facilities to meet residents’ basic health needs in a 15 min healthy community-life circle. Furthermore, we put forward corresponding improvement measures for each health service facility and built a healthy community-life circle with a category and hierarchy system. These findings and suggestions provide some reference for Chengdu and other similar cities to optimize the healthy life circle and have practical significance for improving residents’ health and health service facilities satisfaction.

However, this study also had certain limitations. The high coverage rate of the health service facilities did not mean that residents utilized the facilities within 15 min of walking distance at high speed. Demographic characteristics and population density were also influencing factors. Given that the scale, capacity, service radius, and specific service content of similar health service facilities are difficult to obtain, this work did not consider these factors in the analyses therein, but the health benefits brought by each facility are likely different.

In future studies, the indicators of health service evaluation should not only be medical and health facilities and fitness facilities. More places or environmental elements with health-related behavioral activities, such as entertainment venues, can also be regarded as health-related facilities. In addition, “population density” and “accessibility of other transportation (nonmotorized, public transport, private cars, etc.)” can be taken into account in future studies.

## Figures and Tables

**Table 1:** Classification and weighting of health service facilities in healthy community-life circles.

Category	Item	Content	Total Quantity	Weights
Medical and Health Facilities	General Hospitals	Hospitals with a certain number of beds, separated departments, and corresponding personnel and equipment	1249	0.075
	Specialized Hospitals	Hospitals with only one or a few medical branches, such as cancer hospitals, children’s hospitals, plastic surgery hospitals, etc.	1640	0.025
	Community Hospitals	Provide public health and basic medical services for community members with characteristics of public welfare	201	0.100
	Clinics	Primary medical and health service institutions, no inpatient beds	2514	0.075
	Pharmacies	Facility for daily drug purchase	3101	0.100
Fitness Facilities	Sports Venues	Swimming pools, football fields, basketball courts, badminton courts, table tennis courts, etc.	1120	0.175
	Sports Zones	Places where people play sports in the community, usually small in area	858	0.200
	Parks and Squares	Parks: public green space with facilities and a green environment for the public to visit and carry out physical exercise Squares: open spaces for all kinds of activities	1248	0.250

Note: “Total quantity” is the POI quantity of facilities within the study area.

**Table 2:** Basic profile of the sample (*n* = 371).

	*n*	Weighted (%)
Gender		
Male Female	186 185	50.1 49.9
Age (years)		
Young (18–44) Middle-aged (45–59)	293 56	79.0 15.1
Old (≥60)	22	5.9
Education		
High school degree or below Junior college Bachelor degree or above	102 78 191	27.5 21.1 51.4
Permanent job		
Yes No	248 123	66.8 33.2
Annual income (RMB)		
<50,000 50,000–100,000 >100,000	133 138 100	35.8 37.2 27.0
Type of dwelling		
Low-end Mid-range High-end	60 256 18	16.2 69.0 4.8
Unit/dormitory	37	10.0

**Table 3:** KMO and Bartlett sphere tests.

KMO Test	Bartlett Sphere Test		
	Chi-Square Value	Degree of Freedom	Significant Level
0.861	7711.709	496	0.000

**Table 4:** Descriptive statistics of the resident satisfaction scores.

Accessibility Satisfaction	Mean	SD	Service Satisfaction	Mean	SD
Parks and Squares	3.60	1.276	Parks and Squares	3.43	1.322
Sports Venues	2.94	1.606	Sports Venues	2.91	1.651
Sports Zones	2.99	1.605	Sports Zones	2.87	1.690
General Hospitals	3.17	1.408	General Hospitals	3.17	1.476
Specialized Hospitals	2.82	1.603	Specialized Hospitals	2.76	1.696
Community Hospitals	3.15	1.522	Community Hospitals	3.08	1.546
Clinics	3.28	1.518	Clinics	3.19	1.548
Pharmacies	3.64	1.342	Pharmacies	3.54	1.383
Overall Degree	3.20	1.114	Overall Degree	3.12	1.214

**Table 5:** Ordinal logistic model parameters for satisfaction with accessibility.

		Parks and Squares	Sports Venues	Sports Zones	General Hospitals	Specialized Hospitals	Community Hospitals	Clinics	Pharmacies
Gender	Male	1.131	0.809	0.907	0.886	0.843	0.911	0.870	0.867
	Female	—	—	—	—	—	—	—	—
Age	Young	2.210 *	3.251 **	4.651 ***	2.936 **	2.092	2.445 *	3.755 ***	3.881 ***
	Middle-aged	4.035 ***	2.354 *	4.402 ***	2.273 *	1.868	2.992 **	5.124 ***	7.221 ***
	Old	—	—	—	—	—	—	—	—
Permanent job	Yes	1.718 **	1.697 **	2.069 ***	1.365	1.313	1.473 *	1.590 **	1.788 ***
	No	—	—	—	—	—	—	—	—
Education	High school degree or below	0.937	0.912	1.001	0.661 *	0.847	0.925	1.026	0.986
	Junior college	1.373	0.948	1.556 *	0.897	1.123	1.106	1.254	1.314
	Bachelor’s degree or above	—	—	—	—	—	—	—	—
Annual income (RMB)	<50,000	1.784 **	0.902	1.239	1.142	0.970	1.528	1.379	2.030 **
	50,000–100,000	0.961	0.715	0.944	0.765	1.046	0.951	1.039	0.879
	>100,000	—	—	—	—	—	—	—	—
Type of dwelling	Low-end	0.659	0.887	0.697	0.879	0.991	0.706	0.860	0.610
	Mid-range	0.862	0.736	0.777	0.791	0.757	0.960	0.754	0.811
	High-end	0.596	0.613	0.544	0.770	0.467	0.495	0.298 **	0.397 *
	Unit/dormitory	—	—	—	—	—	—	—	—

Note: The values in the table are OR values. ***, **, and * represent *p* < 0.01, *p* < 0.05, and *p* < 0.10, respectively. When respondents filled out the questionnaire, 1 USD = 6.8148 RMB.

**Table 6:** Ordinal logistic model parameters for satisfaction with service.

		Parks and Squares	Sports Venues	Sports Zones	General Hospitals	Specialized Hospitals	Community Hospitals	Clinics	Pharmacies
Gender	Male	1.011	0.961	1.203	0.999	1.130	0.981	1.077	0.771
	Female	—	—	—	—	—	—	—	—
Age	Young	1.531	3.414 ***	5.624 ***	3.428 ***	3.508 ***	4.450 ***	5.557 ***	2.826 **
	Middle-aged	1.950	1.878	3.232 **	3.149 **	2.866 **	4.459 ***	5.217 ***	4.162 ***
	Old	—	—	—	—	—	—	—	—
Permanent job	Yes	2.102 ***	1.525 **	1.879 ***	1.702 **	1.198	1.174	1.464 *	1.324
	No	—	—	—	—	—	—	—	—
Education	High school degree or below	0.839	1.225	1.411	0.958	1.339	1.046	1.094	0.773
	Junior college	1.112	1.530 *	1.694 **	1.342	1.649 **	1.240	1.448	0.999
	Bachelor’s degree or above	—	—	—	—	—	—	—	—
Annual income	<50,000	2.358 ***	1.373	1.287	1.759 *	1.204	1.621	1.718 *	1.702 *
	50,000–100,000	1.184	1.175	1.023	1.106	0.912	0.870	1.141	0.950
	>100,000	—	—	—	—	—	—	—	—
Type of dwelling	Low-end	0.831	1.242	0.778	1.326	1.017	0.788	0.95	1.033
	Mid-range	0.952	1.250	1.208	1.820 *	0.857	1.065	0.907	1.309
	High-end	0.930	1.358	0.656	1.281	0.512	0.595	0.386 *	0.597
	Unit/dormitory	—	—	—	—	—	—	—	—

Note: The values in the table are OR values. ***, **, and * represent *p* < 0.01, *p* < 0.05, and *p* < 0.10, respectively. When respondents filled out the questionnaire, 1 USD = 6.8148 RMB.

**Table 7:** Regression analysis of satisfaction with accessibility.

	Parks and Squares		Sports Venues		Sports Zones		General Hospitals	
	B	P	B	P	B	P	B	P
Constant		0.000		0.000		0.000		0.000
Frequency	0.066	0.236	0.063	0.240	0.021	0.686	0.008	0.888
Expected distance	−0.261	0.000 ***	−0.174	0.001 ***	−0.252	0.000 ***	−0.194	0.001 ***
Degree of convenience	−0.037	0.500	−0.059	0.259	0.027	0.586	0.105	0.065 *
Walking	0.160	0.012 **	0.248	0.000 ***	0.311	0.000 ***	0.063	0.272
Nonmotor vehicles	−0.045	0.431	0.154	0.004 ***	0.153	0.004 ***	−0.009	0.870
Public transport	0.014	0.810	0.170	0.003 ***	0.175	0.002 ***	0.016	0.788
Private cars	0.036	0.535	−0.007	0.904	0.041	0.458	−0.033	0.590
	**Specialized Hospitals**		**Community−Hospitals**		**Clinics**		**Pharmacies**	
	**B**	**P**	**B**	**P**	**B**	**P**	**B**	**P**
Constant		0.000		0.000		0.000		0.000
Frequency	0.011	0.839	0.114	0.034 **	0.039	0.451	0.009	0.864
Expected distance	−0.202	0.000 ***	−0.238	0.000 ***	−0.323	0.000 ***	−0.382	0.000 ***
Degree of convenience	0.026	0.619	−0.014	0.783	−0.009	0.853	−0.035	0.503
Walking	0.061	0.270	0.221	0.000 ***	0.264	0.000 ***	0.195	0.001 ***
Nonmotor vehicles	0.089	0.098 *	0.091	0.095 *	0.030	0.554	0.040	0.450
Public transport	0.016	0.782	0.068	0.230	0.091	0.085 ***	0.016	0.774
Private cars	0.089	0.121	0.016	0.779	0.063	0.226	0.056	0.321

Note: B: Standardized coefficient. ***, **, and * represent *p* < 0.01, *p* < 0.05, and *p* < 0.10, respectively.

**Table 8:** Paired Sample Tests.

	Correlation of Paired Samples			Paired Sample *t*-Test		
Objectively Measured Average—Perceived Average	N	Correlation	P	Mean	Standard Error	Sig. (2-Tailed)
Parks and Squares	258	0.047	0.453	0.0426	1.821	0.707
Sports Venues	210	−0.085	0.220	−1.3095	1.836	0.000
Sports Zones	227	0.148	0.026	−0.7400	1.556	0.000
General Hospitals	232	0.123	0.061	0.0948	1.733	0.406
Specialized Hospitals	186	0.082	0.264	−1.0806	1.862	0.000
Community Hospitals	224	0.060	0.372	0.7098	1.751	0.000
Clinics	239	0.083	0.198	1.0251	1.709	0.000
Pharmacies	255	0.031	0.628	0.7647	1.609	0.000

**Table 9:** Disordered logistic regression analysis of objective and perceptual measurements of accessibility of and accessibility satisfaction with health service facilities.

		Objective Measurement			Perceptual Measurement		
	Satisfaction Level	OR	95% CI	*p*-Value	OR	95% CI	*p*-Value
Sports Venues	1	0.786	0.37–1.69	0.536	4.727 × 10^−9^		
	2	0.852	0.51–1.43	0.544	0.295	0.17–0.51	0.000
	3	0.866	0.61–1.24	0.427	0.396	0.27–0.58	0.000
	4	1.367	0.91–2.05	0.130	0.520	0.36–0.75	0.001
General Hospitals	1	0.304	0.06–1.61	0.162	0.194	0.04–1.00	0.050
	2	0.520	0.31–0.87	0.013	0.555	0.37–0.84	0.005
	3	1.223	0.90–1.67	0.203	0.585	0.43–0.79	0.001
	4	0.967	0.71–1.31	0.827	0.594	0.44–0.80	0.001
Clinics	1	0.833	0.31–2.23	0.716	1.197 × 10^−9^		
	2	0.668	0.42–1.07	0.093	0.410	0.22–0.75	0.004
	3	0.868	0.67–1.13	0.299	0.525	0.35–0.80	0.003
	4	0.943	0.74–1.20	0.636	0.579	0.39–0.86	0.007

**Table 10:** Ordered logistic regression analysis of objectively measured and perceived measurement accessibility and accessibility satisfaction of health service facilities.

	Objective Measurement			Perceptual Measurement		
	OR	95% CI	*p*-Value	OR	95% CI	*p*-Value
Parks and Squares	1.100	−0.07–0.27	0.272	1.608	0.29–0.65	0.000
Sports Zones	1.006	−0.21–0.23	0.954	1.672	0.30–0.73	0.000
Specialized Hospitals	1.243	0.00–0.43	0.045	1.467	0.18–0.59	0.000
Community Hospitals	1.194	−0.01–0.37	0.065	1.751	0.34–0.78	0.000
Pharmacies	1.089	−0.09–0.26	0.344	1.932	0.42–0.90	0.000

## Data Availability

The raw/processed data required to reproduce these findings cannot be shared at this time as the data also forms part of an ongoing study.
